# Effectiveness of test-and-treat model with direct-acting antiviral for hepatitis C virus infection in migrants: a prospective interventional study in Italy

**DOI:** 10.1186/s40249-024-01200-9

**Published:** 2024-05-28

**Authors:** Nicola Coppola, Loredana Alessio, Stefania De Pascalis, Margherita Macera, Giovanni Di Caprio, Vincenzo Messina, Lorenzo Onorato, Carmine Minichini, Maria Stanzione, Gianfranca Stornaiuolo, Mario Starace, Caterina Monari, Federica Calò, Caterina Sagnelli, Mariantonietta Pisaturo

**Affiliations:** 1 Department of Mental Health and Public Medicine, Section of Infectious Diseases, University of Campania Luigi Vanvitelli, Via: L. Armanni 5, 80131 Naples, Italy; 2 Medical Center, “ Former Canapificio Social Centre “, Caserta, Italy; 3 Medical Center, “Abraham’s Tent” Reception Center, Caserta, Italy; 4 Medical Center, Center for the Protection of the Health of Immigrants, Naples, Italy; 5 Infectious Diseases Unit, AORN Sant’Anna e San Sebastiano, Caserta, Italy; 6 Medical center, Center for the Missionary Sisters of Charity, Naples, Italy

**Keywords:** HCV, Burden, Elimination, Migrant, Italy

## Abstract

**Background:**

Migrants, mainly undocumented and low-income refugees, are at high risk of hepatitis C virus (HCV) infection, but are a difficult-to-reach and to-treat population. The aim of the study was to evaluate the effectiveness of a test and treat model with direct-acting antiviral for HCV infection in these migrants coming from low-income and living in southern Italy.

**Methods:**

A prospective, multicenter, collaborative study based on a four-phase-program (educational counseling, screening, linkage-to-care and treatment) was designed in southern Italy; the study started in June 2018, was stopped in February 2020 because of the outbreak of SARS-CoV2 infection in Italy and was resumed in February 2021 until November 2021.

After educational counseling on infectious diseases that are transmitted through blood or sexually pseudonymized HCV screening was offered to all undocumented migrants and low-income refugees observed at one of the 1st level clinical centers. The HCV-RNA-positive subjects were referred to one of the 3rd level units of Infectious Diseases (ID) and treated with a 12-week course of sofosbuvir-velpatasvir and observed for 12 weeks after the end of direct antiviral agents (DAA) treatment.

**Statistical analysis:**

For the descriptive analysis, the categorical variables were reported as absolute numbers and relative frequencies. Continuous variables were summarized as mean and standard deviation (*SD*) if normally distributed, or as a median and interquartile range (IQR) if not normally distributed. We used Pearson chi-square or Fisher’s exact test for categorical variables and Student’s *t* test or Mann–Whitney test for continuous variables. A *P* value < 0.05 was considered to be statistically significant. Analyses were performed with SPSS 21.0.

**Results:**

Of the 3501migrants observed in the study period, 3417 (97.6%) agreed to be screened; 185 (4.7%) were anti-HCV-positive and, of these, 53 (28.6%) were HCV-RNA-positive. Of these 53 subjects, 48 (90.5%) were referred to an ID unit and started DAA treatment.

The HCV-RNA-positive-subjects were older [median 36 years (IQR: 32–21) vs 27.19 (IQR: 30.5–19.25); *P* = 0.001], and less frequently males [35 (66.03 %) vs 119 (90.1%), *P* < 0 .0001] than seronegative participants. They more frequently came from Eastern Europe (70.8%) stayed longer in Italy [months of stay in Italy, mean ± *SD*: 51.02 ± 52.84 vs 25.7 ± 42.65, *P* = 0.001], and had more years of schooling [years of schooling, mean ± *SD*: 9.61±2.81 vs 7.10 ± 4, *P* = 0.0001]. HCV-RNA-positive-subjects less frequently reported piercing, tattoos and tribal scars as risk factors (23.6%). Of these 48 HCV RNA positive subjects who started DAA, 47 (97.9%) showed a sustained virological response and one dropped-out in follow-up after DAA treatment. No subject had any adverse event.

**Conclusions:**

This model of HCV screening and linkage to care seems effective to eliminate HCV infectionin a difficult-to-reach and to-treat population, such as undocumented migrants and low-income refugees. The participation of cultural mediators in the study made possible a better interaction between migrants and physicians, as is evident from the large number of subjects enrolled. Eliminating HCV among migrants will have a long-term positive impact from a public health and healthcare perspective by reducing the number of individuals who potentially develop HCV-related complications such as liver cirrhosis and hepatocellular carcinoma and reducing the circulation of HCV in the regions that host them which often, as in the case of Italy, are low endemic for HCV infection.

**Graphical Abstract:**

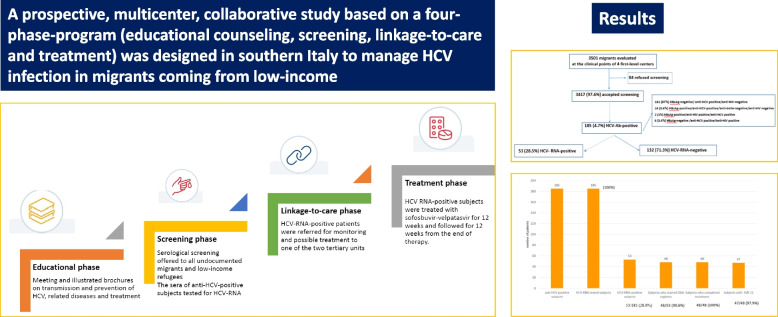

**Supplementary Information:**

The online version contains supplementary material available at 10.1186/s40249-024-01200-9.

## Background

The hepatitis C virus (HCV) is a blood-borne virus and most infections occur through exposure to blood from unsafe health-care procedures, unscreened blood transfusions, injection drug use and sexual practices. If left untreated, HCV infection can result in complications such as liver cirrhosis, hepatocellular carcinoma, liver failure, and even death.

Polaris Observatory HCV Collaborators estimated a global prevalence of viraemic HCV infection of 0.7% (95% Uncertainty Interval: 0.7–0.9), corresponding to 56.8 million (95% Uncertainty Interval: 55.2–67.8) infections, on January 1, 2020 [1]. Data on HCV prevalence in Italy are scanty and fragmented. Some reports estimated in Italy an HCV prevalence range from 0.7% to 1.3% [1, 2].

Interferon-free treatment with direct-acting antivirals (DAAs) provides an excellent opportunity to prevent the progression of liver disease [3] and the global elimination of HCV has become a realistic goal as suggested by the WHO. However, although DAAs can cure more than 95% of persons with hepatitis C infection, access to diagnosis and treatment is low [4, 5].

The WHO suggests developing and implementing HCV elimination strategies both in specific populations at high prevalence and/or at high risk for HCV infection such as migrants, men who have sex with men, people who inject drugs, prisoners and in the general population. They have set goals that HCV could be eliminated within 2030 (i.e. 90% reduction in new chronic infections and 65% reduction in mortality) [1, 3]. Several programs of HCV case-finding and linkage-to-care have been carried out in specific populations at high prevalence and/or at high risk for HCV infection, such as individuals living with human immunodeficiency infection (HIV), hemophilia, injection drug users and prisoners [3, 6, 7]. The control of HCV infection in these populations allows the control of HCV infection also in the general population.

Migrants born in intermediate and high HCV-prevalence countries who live in low HCV- prevalence countries are likely to be at an increased risk for HCV infection due to exposure in their countries of origin [8].

Greenaway et al. [9] in a meta-analysis on 50 studies including 38,635 migrants from all world regions estimated an overall anti-HCV prevalence (previous and current infections) of 1.9% [95% confidence interval (*CI*): 1.4–2.7%).

Italy is a landing place for migratory flows, mostly moving from sub-Saharan Africa: for example, in 2022, Italy, in particular southern Italy, was reached by about 100,000 migrants (last updated 25 December 2022) [10]. Data on HCV prevalence in migrants living in Italy are scanty and contrasting. In a cohort of 3,720 migrants coming from non-EU and non-western countries (USA, Canada, and Australia) living in Northern Italy the anti-HCV prevalence was 3.6% [11] while in a cohort of 2639 African migrants living in Sicily, Southern Italy, the anti-HCV prevalence was of 0.9% [12].

Despite a possible considerable prevalence of HCV infection in this population, there are few screening and linkage-to-care programs for this target.

Therefore, in the present study we propose an innovative model to detect and to eliminate HCV infection in undocumented migrants and low-income refugees to contribute to achieving the WHO goal of eliminating HCV infection by 2030.

HCV elimination in migrants will reduce the risk of complications related to chronic infection in this target population; this will have a great benefit ensuring the reduction of HCV burden also on Public Health of the hosting country

## Methods

### Study design

A prospective, multicenter, collaborative study, based on the long-term active cooperation between two 3rd level units of Infectious Diseases and four 1st level clinical centers in southern Italy (Naples and Caserta – in the Campania region) was designed [13–17]. The study started in June 2018, was stopped in February 2020 because of the outbreak of SARS-CoV2 infection in Italy and was resumed in February 2021 until November 2021

The cities involved in the study host a large migrant population coming from low-income countries, especially from western Africa, middle and eastern Asia and Eastern Europe. Migrants from Africa move for economic reasons, to escape war and persecution, or to reunite with their family members. Migrants from Eastern Europe move for economic reasons or to reunite with their relatives [13–17].

The first-level clinical centers are general practice clinics that are attended mainly by migrants for low back pain, headache, itching, cough, hypertension and allergy symptoms; thus, they have proven experience in managing vulnerable groups and are greatly appreciated by the migrants. These first-level centers are linked with the Italian humanitarian organizations "La Caritas" the Tent of Abraham" and "Emergency", which welcome migrants who need help, offering refuge even if temporary, hot meals, and medical and legal assistance. The migrants willingly frequent these associations because they know they can find help, they know they can find competent people who try to help them obtain temporary documents, in order to find work and to join their families in other European countries [13–17].

Moreover, the present study program was facilitated by the work of cultural mediators, professional who facilitates the communication, including interpretation, between people speaking different languages and coming from different cultural backgrounds [18] and was a fundamental link between the physicians and the migrants.

By the term “migrant” as that “a person living in another country than he/she is born” and we refer to a heterogeneous population including undocumented migrants (persons moving outside regular migration channels), asylum seekers (individuals whose are seeking international protection for fleeing persecution or serious harm or for other reasons), refugees (people fleeing persecution or conflicts seeking international protection under the 1951 Refugee Convention on the Status of refugees) and economic migrants (people whose primary motivation for fleeing their home country is to improve their economical situation) [18].

We included both newly-arrived migrants and subjects who had been living in Italy for more than one year. We considered as “newly-arrived migrants” subjects living in Italy less than one year and as “anciently-arrived migrants” subjects living in Italy for more than one year.

Refugees have access to all the healthcare facilities of the National Health System; for undocumented migrants access is limited to minors, pregnant women and patients with severe pathological conditions or communicable diseases.

### Phases of the study

**Fig. 1:**
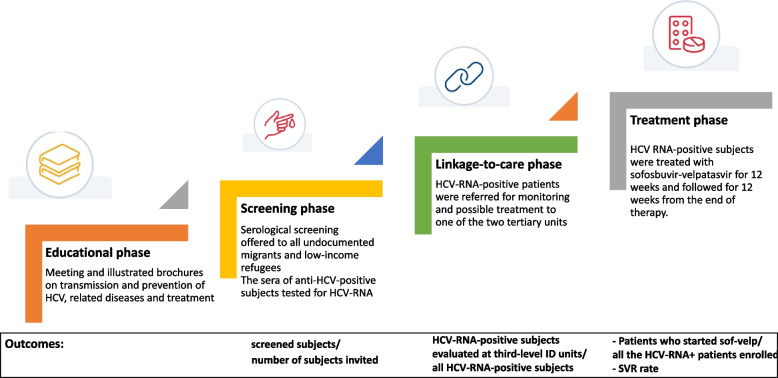
Phases of the study and outcomes

All ≥ 18 years old consecutively evaluated for clinical or legal consultation at one of the four first-level centers were enrolled in the present study program, organized in four phases (Fig. 1). Thanks to the collaboration between the cultural mediator and the physicians and nurses, all the subjects evaluated at the first level centers in the study period were offered to participate in the study.

The first part of the study included an "educational" phase: the physicians, with the help of cultural mediators, explained to the migrants who attended the first-level centers what were the main sexually and blood transmitted infectious diseases endemic in their countries of origin and information on the routes of transmission. The physicians indicated that the use of condoms protected them from sexually transmitted diseases and the use of personal sharp objects was a risk for blood transmitted infectious diseases; moreover, the natural history of HCV infection and of the other parenteral infectious diseases and the availability of very effective and safe antiviral therapy were explained. The information/education was done through meetings and using brochures with pictures and explanations translated into English, French and Swahili.

After the educational phase, at first-level medical centers pseudonymized serological screening (recording only the center number and patient's number) was offered to seek HIV, HBV and HCV in full accordance with the Italian privacy law regarding observational studies (second phase). The migrants, who agreed to join the study, signed an informed consent written in the immigrant's own language and filled out a pseudonymized questionnaire administered by the research investigators with the assistance of a cultural mediator on the demographic data (age, gender, race/ethnicity, place of birth, language), date (month and year) of immigration, socioeconomic status (level of education), religion, cohabitation details, sexual orientation and practices including condom use, history of HBV vaccination, surgery, dental care, tattooing, body piercing, use of drugs, blood transfusion, tribal rituals, abortion and information on previously documented personal and family infections of HBV, HCV and HIV. The data relating to the epidemiological characteristics were collected in an electronic database. The clinical history was obtained with the help of a physician and a cultural mediator during a prolonged, in-depth clinical consultation and counseling.

All subjects included in the study were screened for hepatitis B surface antigen (HBsAg), anti-HCV and anti-HIV.

The sera of HBsAg-positive subjects were tested for serum HBV DNA anti-delta (data not shown). The sera of anti-HCV-positive subjects were tested for HCV-RNA.

The third part of the study included a "linkage-to-care" phase: the participants who were positive for HCV-RNA were referred for monitoring and possible treatment to one of the two tertiary units of infectious diseases operating in the same city. Obviously, also HBsAg and/or anti-HIV-positive subjected were linked-to-care to one of the two tertiary units participating in the study (data not shown). Also in this phase, cultural mediators played an important role because they accompanied migrants to third level centres . At the third-level infectious disease units, all the HCV-RNA-positive subjects were tested for HCV genotype.

The last phase of the present study program was the “treatment” phase. All the HCV-RNA-positive subjects were offered antiviral treatment. Migrants who agreed were treated with sofosbuvir-velpatasvir (Gilead Science) for 12 weeks and followed-up for 12 weeks until the end of therapy. The choice of sofosbuvir-velpatasvir was based on the use of a pan-genotype regimen with the same schedule both in cirrhotic and non-cirrhotic patients. Response to antiviral treatment was defined as sustained virological response (SVR: HCV-RNA undetectable) 12 weeks (SVR12) after the end of therapy. Relapse was defined as a reappearance of serum HCV-RNA after DAA treatment.

### Outcomes of the study

The end-points of the different phases of the present study were: for the screening phase, the proportion of screened subjects for HBV, HCV and HIV versus the number of evaluated subjects in the period study.

For the linkage-to-care phase, the proportion of HCV-RNA-positive participants evaluated at third-level infectious disease units versus all anti-HCV positive subjects; for the treatment phase, the proportion of HCV-RNA-positive-patients who started sofosbuvir-velpatasvir treatment versus all the HCV-RNA-positive-patients enrolled, and the SVR rate by an intention-to-treat analysis.

### Ethics statement

The study was approved by the Ethics Committee of the Azienda Ospedaliera Universitaria of the University of Campania Luigi Vanvitelli, Naples, Italy (214/2012; 481/2018). All procedures performed in this study were in accordance with the ethics standards of the institutional and/or national research committee and with the 1964 Helsinki declaration and its later amendments or comparable ethics standards.

This work was in part supported by Gilead, who supplied sofosbuvir/velpatasvir.

### Testing procedures

Serum samples were tested for HBsAg, total anti-HCV, anti-HIV, anti-HBc and hepatitis B surface antibodies (anti-HBs) by commercial immunoenzymatic assays [Abbott Laboratories, North Chicago, IL, United States: AxSYM HBsAg (V.2) M/S for HBsAg, AXSYM HCV 3.0 for anti-HCV, AXSYM HIV 0.5 COMBO for anti-HIV, AXSYM core for anti-HBc and AXSYM AUSAB for anti-HBs]. Anti-HIV reactivity was always confirmed by a Western blot assay (Genelabs Diagnostics, Science Park Drive, Singapore), which identifies both HIV-1 and HIV-2 strains. Anti-delta was tested in HBsAg-positive subjects by anti-HDV Elisa (Dia.pro diagnostic bioprobes, Sesto San Giovanni, Milano, Italy).

Serum HCV-RNA was quantified by a real-time PCR method in a Light cycler 1.5 (Roche Diagnostics, Branchburg, NJ, USA) with a limit of detection < 40 IU/ml, as previously reported [19, 20]. HCV genotypes were identified by the HCV genotype Lipa assay (Bayer, Lyon Cedx 09, France).

### Statistical analysis

For the descriptive analysis, the categorical variables were reported as absolute numbers and relative frequencies. Continuous variables were summarized as mean and standard deviation (*SD*) if normally distributed, or as a median and interquartile range (IQR) if not normally distributed.

We used Pearson chi-square or Fisher’s exact test for categorical variables and Student’s *t* test or Mann–Whitney test for continuous variables. A *P* value < 0.05 was considered to be statistically significant. Analyses were performed with SPSS 21.0 (IBM, Armonk, NY, USA).

## Results

### Epidemiological characteristics of study population

**Fig. 2:**
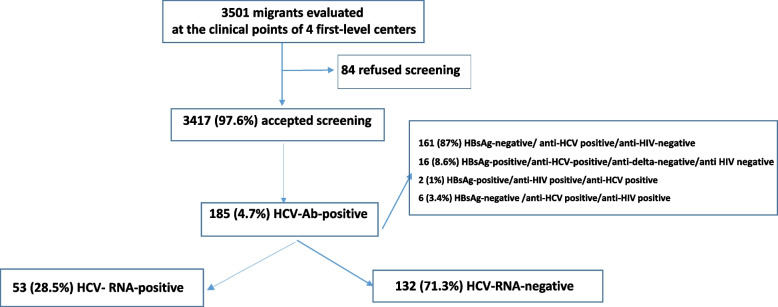
Flow chart of the screening of the subjects at the first clinical level centers

Three thousand five hundred one consecutive visitors with a migration background had visited the first-level centers during the indicated periods; 3417 (97.6%) agreed to be screened (Fig. 2). Supplementary Table shows the characteristics of the 3417 subjects screened.

They were mainly males (61%), with a median age of 27 years (IQR: 8–74). The average number of months spent in Italy was 28.3 (± 45.1). As regards the geographical area of origin, 2,066 subjects (58%) were from sub-Saharan Africa (especially from Ghana and Senegal with 488 and 480 subjects, respectively, data not shown in Supplementary Table), 642 (19%) from Asia (especially from Pakistan and Bangladesh with 251 and 239 subjects, respectively data not shown in Supplementary Table), 310 (9%) from Eastern Europe (especially from Ukraine and Bulgaria with 160 and 118 subjects, respectively, data not shown in Supplementary Table), 141 (4%) from northern Africa, 34 (0.4%) from South America; for 224 (1%) the geographical origin was not known because subjects preferred not to answer.

### Results of the virological screening

Of the 3417 subjects screened 185 (4.7%) were anti-HCV-positive (Fig. 2). The complete serological status of the 3417 subjects screened is shown in Supplementary Table. None of the patients enrolled was aware of their serological status and none of the enrolled subjects had any knowledges on viral hepatitis, on the mode of transmission of these viruses and on the epidemiology of these infections in their countries of origin.

Table 1 shows the epidemiological characteristics of the 3417 subjects enrolled according to serum HCV status.

**Table 1:** Epidemiological characteristics of the 3417 subjects enrolled according to serum HCV status

	**Anti-HCV-positive**	**Anti-HCV-negative**	***P* value**
**Subjects (*N***^a^**)**	185	3232	
**Age, years, median (IQR)**	31.5 (32–21)	27 (30.5–19.25)	0.001
**Males, *N***^a^** (%)**	148 (82.2)	2544 (78.7)	0.288
**Months of stay in Italy, (mean ± *SD*)**	32.4 ± 47.64	30.24 ± 46.36	0.005
**Years of schooling (mean ± *SD*)**	8.2 ± 3	6.26 ± 4.67	0.000
**Country of origin**^a^**, *n*/*N* (%)**			
** Sub-Saharan Africa (2107 subjects)**	79/2107 (3.9)	2028/2107 (96.1)	< 0.000
** Eastern Europe (316 subjects)**	22/316 (6.9)	294/316 (93.0)	0 .201
** India-Pakistan (490 subjects)**	25/490 (5.1)	465/490 (94.8)	0.711
** North Africa (136 subjects)**	1/136 (0.73)	135/136 (99.2)	0.023
** Others (242 subjects)**	58/242 (23.9)	184/242 (76.0)	< 0.000
**Risk factors**^a^**, *n*** ^a^** (%)**			
** Drug addiction**	5/19 (26.3)	14/19 (73.6)	0.000
** Sexual intercourse without a condom**	71/1489 (4.7)	1418/1489 (95.2)	0.142
** Surgical interventions, dental procedures, Intramuscular therapy**	96/2274 (4.2)	2178/2274 (95.7)	0.000
** Tattoo, piercing, tribal scars**	38/746 (5.09)	708/746(94.9)	0.661
**Serum status for other viral infections, *N***^a^** (%)**			
HBsAg-positive, anti-delta/anti-HIV-negative	16 (8.6)	300 (9.2)	0.772
HBsAg/anti-delta-positive, anti-HIV-negative	0	8	-
HBsAg positive/anti-HIV positive	2 (1)	8 (0.2)	0.799
HBsAg negative / anti-HIV positive	6 (3.2)	60 (1.85)	0.182

-: Not applicable

*HCV* Hepatitis C Virus virus, *IQR* Interquartile range, *SD* Standard deviation

^a^% for line

The 185 anti-HCV-positive subjects were older than the 3232 anti-HCV negative subjects [31.5 (IQR: 32–21) vs 27.5 (IQR: 30.5–19.25) years; *P* = 0.001]. Males were the majority in both groups. The anti-HCV-positive subjects had been in Italy for longer time (mean ± *SD*: 32.4 ±47.64 vs 30.24 ± 46.36 months, *P* = 0.005) and had more years of schooling (mean ± *SD*: 8.2 ± 3 vs 6.26 ± 4.67, *P* = 0.000). The anti-HCV-positive-subjects more frequently than the anti-HCV-negative were from Eastern Europe (6.9%) in particular 8.75% (14/160) came from Ukraine, 12.5% (2/16) from Romania, 5% (6/118) from Bulgaria.

Anti-HCV-positive subjects were more frequently intravenous drug addicts (26.3%). There were not differences in risk factors between anti-HCV-positive males and females (data not shown).

In the anti-HCV-positive group, 16 (8.6%) had concomitant HBV infection, 6 (3.2%) had concomitant HIV infection and 2 (1%) had concomitant both HBV and HIV infection (Table 1). However, there was no difference in the prevalence of other viral infections between the two groups.

All the 185 anti-HCV positive subjects agreed to be identified for the subsequent virological investigations.

Of these 185, 53 (28.7%) were HCV-RNA-positive and 132 (71.3%) negative (Fig. 2). The 132 anti-HCV-positive/HCV-RNA-negative subjects were re-tested for HCV-RNA at a first-level medical center and all resulted HCV-RNA-negative again; none reported a previous antiviral treatment. Table 2 shows the epidemiological characteristics of the 185 anti-HCV-positive subjects according to HCV-RNA-positivity.

**Table 2:** Epidemiological characteristics of the 185 anti-HCV-positive subjects according to HCV- RNA-positivity

	**HCV-RNA-negative subjects**	**HCV-RNA-positive subjects**	***P* value**
**Subjects, *N***^a^	132	53	
**Age, years median (IQR)**	27.19 (30.5–19.25)	36 (32–21)	0.0001
**Males, *N***^a^** (%)**	119 (90.1)	35 (66.03)	< 0.0001
**Country of origin**^a^**, *N***^a^** (%)**			
** Sub-Saharan Africa (76 subjects)**	62/ 76 (81.5)	14/76 (18.4)	0.010
** Eastern Europe (24 subjects)**	7/24 (29.1)	17/24 (70.8)	< 0.00001
** India-Pakistan (26 subjects)**	18/26 (69.2)	8/26 (30.7)	0.796
** North Africa (1 subject)**	0/1	1/1 (100)	-
** Others (58 subjects)**	45/58 (77.5)	13/58 (22.4	0.204
**Months of stay in Italy, mean ± *SD***	25.7 ± 42.65	51.02 ± 52.84	0.0012
**Years of schooling, mean ± *SD***	7.10 ± 4	9.61 ± 2.81	0.0001
**Risk factors**^a^**, *N***^a^** (%)**			
** Drug addiction**	3/5 (60)	2 /5(40)	0.5692
** Sexual intercourse without a condom**	55/71 (77.4)	16/71 (22.5)	0.146
** Surgery, dentistry, abortions and transfusions**	72/96 (75)	24 /96(25)	0.2542
** Piercings, tattoos, tribal scars**	29/38 (76.3)	9/38 (23.6)	< 0.00001

-: Not applicable

*HCV* Hepatitis C Virus virus, *IQR* Interquartile range, *SD* Standard deviation

^a^% for line

The HCV-RNA-positive-subjects were older [median 36 years (IQR: 32–21) vs 27.19 (IQR: 30.5 –19.25); *P* = 0.001], and less frequently males [35 (66.03 %) vs 119 (90.1%), *P* < 0 .0001] than seronegative participants. They more frequently came from Eastern Europe (70.8%) stayed longer in Italy (months of stay in Italy, mean ± *SD*: 51.02 ± 52.84 vs 25.7 ± 42.65, *P* = 0.001), and had more years of schooling, mean ± *SD*: 9.61 ± 2.81 vs 7.10 ± 4, *P* = 0.0001). HCV-RNA-positive-subjects less frequently reported piercing, tattoos and tribal scars as risk factors (23.6%).

### Results of the linkage to care phase

In the third phase of the program (linkage-to-care phase), of the 53 HCV-RNA-positive-subjects, 48 (90.5%) were referred for monitoring and possible treatment to one of the two tertiary units of infectious diseases and 5 refused the linkage to care. Of these 48, 16 (33.3%) harboured HCV genotype 1b, 11 (22.9%) genotype 1a, 16 (33.3%) genotype 3, 3 (6.3%) genotype 4 and 2 (4.2%) genotype 2. No subjects with chronic hepatitis had biological markers of liver cirrhosis.

**Fig. 3:**
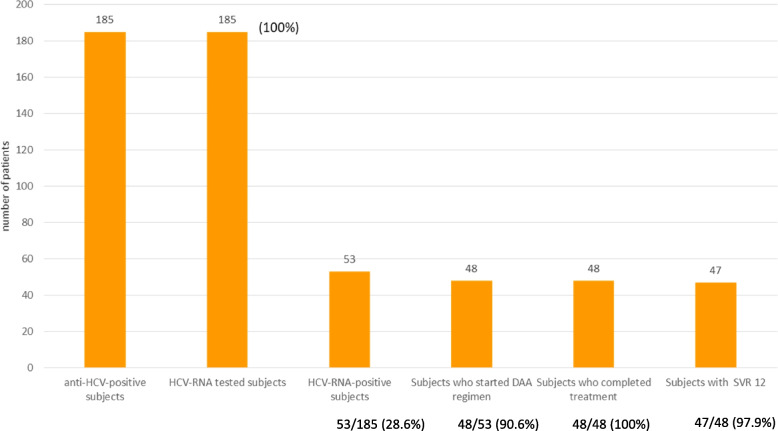
Cure-cascade of anti HCV positive subjects linked to third clinical level centers, HCV: hepatitis C virus

All the 48 HCV-RNA-positive patients started DAA-regimen with sofosbuvir plus velpatasvir and completed the 12 weeks of treatment. Of these 48 subjects, 47 (97.9%) showed a sustained virologic response (SVR) at 12 and at 24 weeks after treatment and one dropped-out in follow-up after finishing the DAA treatment. No subject had any adverse event. Figure 3 shows the HCV-cascade of the present program.

In conclusion, all 185 anti-HCV positive-subjects were tested for HCV-RNA; of the 53 HCV-RNA-positive, 48 (90.5%) were linked to an infectious disease unit and started DAA regimen; of these 48, 47 (97.9%) showed SVR (Fig. 3).

## Discussion

Interferon-free treatment with DAAs provides excellent chances for HCV elimination [3]. The WHO set as goal the elimination of viral hepatitis by 2030; among the target populations that needed screening, linkage-to-care and treatment programs, there were also individuals originating from a country with an HCV prevalence of > 2% [8].

Migrants lack access to optimal healthcare services due to different types of barriers, such as patient-physician communication, language problems, legal and bureaucratic barriers, beliefs in traditional medicine, ethnic disparities and inadequacies arising from socio-economic problems including a lack of family support [21]. On arrival at reception areas, infectious diseases are not a health priority for migrants. Moreover, migrants do not always have adequate knowledge of HCV infection, its evolution and treatment [22] and therefore remain undiagnosed and untreated [23].

With the present model we enrolled about 3500 migrants from areas endemic for hepatitis virus infections. After an educational phase on the route of transmission, natural history and treatment availability for HCV infection, nearly 98% of subjects agreed to be tested for hepatitis virus infections, about 91% of the HCV-RNA-positive patients were linked to care at one of the infectious disease units and started DAA treatment, and all but one obtained a sustained virological response. It was possible to perform the HCV RNA and HCV genotype because our study won a funding from Gilead Science which also provided the DAAs used for the treatment of the enrolled subjects free of charge.

Thus, the program achieved outstanding success, probably because it was performed directly where the migrants were, that is, in humanitarian organizations, with free access even for migrants who did not have valid identification documents. These humanitarian organizations meet the basic needs of migrants (hot meals, and medical and legal assistance), and thus allowed us to enroll and link to care a difficult-to-manage population. In fact, one of the main problems of migrants was the lack of valid documents and these associations helped them, through legal tutelage, to get these valid documents, which allowed them to leave their undocumented status and to become part of the community, only in this way could they hope to find work, improve their socio-economic condition and approach our national health service. For all these reasons the migrants willingly attended these associations and the rate of acceptance of the different phases of the program, from HCV screening to follow-up during and after DAA regimens, was very high.

Another key-point of the present program was the role of cultural mediators, who helped us gain the trust of the migrants and to overcome the bureaucratic, cultural and social problems in allowing HCV screening and managing infection in HCV subjects.

Specifically, our program consisted of four different phases. First, a phase of information and education on sexually transmitted infectious diseases and a subsequent phase of HCV screening. Informing migrants that there are sexually and blood transmitted ly infectious diseases widespread in their countries of origin made them aware that they could have contracted one of these infectious diseases. All this was demonstrated by the high percentage of subjects who agreed to be screened (about 98%). Most of them were unaware of the existence of infectious diseases potentially at risk to their health and did not know that there were treatments that would have avoided serious problems for their health. Furthermore, screening was free of charge and pseudonymized and this was an additional incentive to make it easier for them to accept.

Interestingly, only 30% of anti-HCV-positive subjects were HCV-RNA-positive in the present study.

These data can be explained by the fact that they come from a population-based study, and not from analysis performed in liver units. Thus, the data of the present study may be useful to clarify the prevalence of HCV in the general population of migrants and a warning for the Italian Healthcare Authorities to develop suitable cost-effective screening policies in this setting.

There are no standardized screening programs for infectious diseases in migrants worldwide: for example, in the US, the immigration medical examination does not include tests for viral hepatitis [23]; testing for viral hepatitis is not mandated also in Canada, even in patients originating from countries with high prevalence [24]; in the EU region, instead, the immigration medical screening policies are country-specific [25]. Even in Italy there is no screening program organized by the Italian national health system but there are initiatives by individual clinical research groups that deal with the screening of migrants. In these experiences the prevalence of anti-HCV-positive-migrants varied from 0.9% to 20% [11, 12, 16].

In the third and fourth phases, the HCV-RNA-positive patients were linked to care at one of the Infectious Disease units participating in the study and were treated with sofosbuvir plus velpatasvir for 12 weeks. We obtained a linkage-to-care percentage of almost 90% and an SVR at an intention to treat analysis of about 98%.

In line with our results, Prestileo et al investigating 2639 migrants arrived in Sicily, Southern Italy, showed 24 (0.9%) anti-HCV-positive; of these 24, 18 subjects were HCV RNA positive and 11 (61%) received DAA therapy, 10 (90%) of them completed therapy and achieved sustained virological response, and one patient was lost to follow-up after 4 weeks of therapy [12].

Undiagnosed and untreated cases of viral hepatitis among migrants are important points that favor the growing morbidity and mortality. It is known that without treatment, approximately 20% of people with chronic HCV infection progress to liver cirrhosis, which can lead to end-stage liver disease and the development of HCC [26], with important implications for the host country's healthcare system.

Moreover, despite the availability of effective therapies, DAA based regimens were only moderately cost-effective and as a result less than 30% of people with HCV had been screened and less than 5% of all HCV cases had been treated in the EU/EEA in 2015. Migrants had additional barriers in linkage to care and treatment due to several health system barriers [9].

Few studies have been published at present on the care and DAA-treatment outcome of HCV-positive migrants due to the scanty possibility of screening, late submission to a clinical center authorized for the free administration of drugs and poor adherence to treatment and follow-up. For example, in the Canadian Network Undertaking against Hepatitis C (CANUHC), including 725 HCV-infected patients assessed for DAA treatment (18.5% were born outside the country), there was a similar proportion of subjects initiating DAA therapy (56.7% vs 49.9%) and SVR rates (89.4% vs. 92.5%) among migrants and native subjects [27]. In another study conducted in Spain on 175 immigrant patients treated with DAAs, an SVR was obtained in 156 (89.1%) [28]. Yasseen et al [29], including 940,245 individuals, estimated an hepatitis C prevalence of 0.7% in all immigrants and 1.1% in immigrants coming from HCV endemic countries; of the 14,802 anti-HCV-positive individuals, 11,290 had follow-up nucleic acid tests conducted, 6731 were HCV RNA positive, 2319 individuals were on treatment and only 986 (42%) showed evidence of viral clearance. However, it seems to be important to underline that many subjects in this study were treated with less effective therapeutic regimens, such as the combination of interferon and ribavirin, while our subjects were all treated with DAAs; this explains the lower prevalence of SVR in the Canadian cohort compared to our cohort.

### Limitations

Our model is easily reproducible from a methodological point of view but has limitations.

The main problem is certainly represented by the difficulty in finding funds to carry out screening, linkage to care and to provide therapy to populations with limited economic resources such as migrants from low -income countries.

Our study was carried out thanks to Gilead Science which offered the antiviral treatment free of charge and made a financial contribution to carry out the screening.

Another fundamental point is the right choice of cultural mediators who have a fundamental role: bringing migrants closer to physicians, facilitating a bond of trust and following them throughout the screening and treatment process.

## Conclusions

Our model of HCV screening and linkage to care in target populations is effective in the HCV screening, linkage-to-care and treatment in a difficult-to-reach and to-manage population, such as undocumented migrants and refugees. In the last three years the COVID-19 pandemic has had a deep impact on the management of chronic liver diseases, which will likely postpone the WHO goal of HCV eradication by 2030 [30]. It is necessary to continue with HCV screening and treatment in target populations and in the general population thus ensuring the achievement of the WHO goal of eradicating HCV in 2030.

Eliminating HCV among migrants will have a long-term positive impact from a public health and healthcare perspective by reducing the number of individuals who potentially develop HCV-related complications such as liver cirrhosis and hepatocellular carcinoma and reducing the circulation of HCV in the regions that host them which often, as in the case of Italy, are low endemic for HCV infection.

### Supplementary Information

**Supplementary material 1.**

## Data Availability

The data can be requested from the corresponding author, Prof Mariantonietta Pisaturo (mariantonietta.pisaturo@unicampania.it)

## Abbreviations

HCV: Hepatitis C virus
ID: Infectious Diseases
*SD*: Standard deviation
IQR: Interquartile range
DAA: Direct antiviral agents
IU: International Unit
*CI*: Confidence interval
