# Supplementary material for: Effectiveness of test-and-treat model with direct-acting antiviral for hepatitis C virus infection in migrants: a prospective interventional study in Italy
Source: Infect Dis Poverty. 2024 May 28;13:39. doi: 10.1186/s40249-024-01200-9 (PMC11131219; doi:10.1186/s40249-024-01200-9)
Supplement: Supplementary file 1 — Supplementary material 1. [file 40249_2024_1200_MOESM1_ESM.docx]

**Supplementary Table 1:** Demographic and serological characteristics of the 3417 migrants enrolled

| **Number of patients** | 3417 |
| --- | --- |
| **Age, median (IQR)** | 27 (18-74) |
| **Males, n ° (%)** | 2805 (61) |
| **Months of stay in Italy, median (SD)** | 28.3 (±45.1) |
| **Geographical area of origin n ° (%)**  Eastern Europe  India-Pakistan  North Africa  Sub-Saharan Africa  South America  Not known | 310 (9,07)  642 (18.7)  141 (4.12)  2066 (60,4)  34 (0.99)  224 (6,5) |
| **Serological marker n° (%)**  HBsAg-positve, anti-HCV-negative, anti-delta-negative, anti HIV-negative  HBsAg negative/ anti-HCV positive/anti-HIV-negative  HBsAg-positive, anti-HCV-positive, anti-delta-negative, anti HIV negative  HBsAg-positive, anti-delta-positive, anti-HCV-negative, anti HIV negative  HBsAg-negative, anti-HBc-positive, anti HIV negative, anti HCV negative  HBsAg positive/anti-HIV positive/anti-HCV negative  HBsAg positive/anti-HIV positive/anti-HCV positive  HBsAg negative /anti-HCV positive/anti-HIV positive  HBsAg negative/ anti- HIV positive/anti-HCV negative  HBsAg /anti-HCV /anti-HIV/anti-HBc negative | 300 (8.7)  161 (4.7)  16 (0.5)  8 (0.2)  1332 (39)  8 (0.2)  2 (0.05)  6 (0.1)  60 (1.7)  1524 (45) |
